# Income, Demographics, and Life Experiences of Clinical-Forensic Psychologists in the United States

**DOI:** 10.3389/fpsyg.2022.910672

**Published:** 2022-07-07

**Authors:** Tess M.S. Neal, Emily N. Line

**Affiliations:** ^1^New College of Interdisciplinary Arts and Sciences, Arizona State University, Phoenix, AZ, United States; ^2^School of Psychology, University of New South Wales, Sydney, NSW, Australia; ^3^Department of Psychology, University of Illinois Urbana-Champaign, Champaign, IL, United States

**Keywords:** gender, psychology, women, forensic, professional equality, income, hourly rate, salary

## Abstract

We provide aggregate data about income, demographics, and life experiences of women and men practicing clinical-forensic psychology primarily in the United States (*N* = 376). We examine how these variables relate to one another, as well as how gender demographics of the field have changed over time. The average hourly rate charged by psychologists for forensic work, aggregated across all types of referral questions, regions, and employment settings is $280.23 (US Dollars; SD = $108.12; median and mode = $250). Total median annual income is = $125,000 - $149,999 and mode is = $100,000 - $124,999. Men’s annual income (median = $175,000 - $199,000) is significantly higher than women’s (median = $100,000 - $124,999) even while controlling for years of experience and number of hours worked per week. Women forensic psychologists earn $0.83 for every $1.00 men make. Having a Ph.D. is disproportionately associated with men and PsyD with women; however, the difference is not significant once controlling for years of experience. Employment type related to pay, such that people in private practice make significantly more than those who work in institutions (e.g., prisons, hospitals) or universities. Year of highest degree associated with employment type, such that people in practice longer are more likely to be in private practice. Although we expected caretaking responsibilities and children would relate to gender and pay, no differences emerged in this sample. Women are more likely than men to have completed a formal postdoctoral fellowship in forensic psychology, even when controlling for year of highest degree. Regarding the gender composition of the field over time, we calculated the Index of Dissimilarity for each five year increment spanning 1965-2019. Before the late 1990s, proportionally more men entered the field; after the late 1990s, proportionally more women entered. We discuss the promising and less promising implications of these findings for gender equity and work-life management in forensic psychology, as well as how professionals in the field and students might make use of these data.

## Introduction

“*I have advocated*…*basic rights which I believe every citizen in a democracy must enjoy. These [include]*…*the right to work for equal pay according to ability*.”

– Eleanor Roosevelt, 1944

Eleanor Roosevelt, lifelong human-rights advocate and former First Lady of the United States, championed the cause of equality and women’s meaningful participation in society. In her last public position before her death, she served as chair of President John F. Kennedy’s Presidential Commission on the Status of Women (1965), which documented the status of women in America, criticized the inequalities they faced, and offered recommendations for change. She prefaced the report, suggesting many of these barriers to women’s ambitions would disappear in the near future as women’s place in society improved.

Indeed, much progress has been made toward professional equality for men and women in the last half-century since that report, introducing changes to American society from all three branches of U.S. government (i.e., new laws passed by Congress, executive orders issued by Presidents, and decisions by the Supreme Court). Women and men are represented in increasingly equal numbers throughout higher education and in most occupations. According to the National Science Foundation [NSF] (2021), women now receive 57.4% of all bachelor’s degrees awarded to undergraduate students and they earn 50.4% of all doctoral degrees conferred. Further, averaged across all jobs, women working full-time in the United States are now earning $0.82 for every $1.00 men make compared to the $0.62 they made in 1979 (Bureau of Labor Statistics, 2021)^1^.

Despite various indicators of progress, patterns of real differences between men and women in earnings, job satisfaction, and advancement potential have endured, and in some cases have gotten worse. In psychology, historical gaps have closed for several indicators of parity at the early career level (e.g., hiring decisions, initial promotions, attainment of research grants, early career-awards), though disparities still remain in many areas and persist even in these particular indicator types at more senior levels (Gruber et al., 2021). Still today, women psychologists are paid less, achieve less career success and eminence, are less productive (by various metrics), are less likely to enter academic tenure-track positions, and are asked to spend more time on service than men (Gruber et al., 2021).

Understanding gender parity in income and other indicators of occupational health can inform how to eliminate those gaps and improve a field. These indicators are worth examining particularly in the forensic psychology subfield, as variation across subfields of psychology is likely (Gruber et al., 2021), and forensic psychologists face unique benefits and challenges (Neal, 2018). Income equity has tangible benefits, such as improved employee engagement and performance (Noland et al., 2016), and when compensation information is available, gender pay gaps are smaller (American Association of University Women [AAUW], 2022). We consider stable gender integration with roughly equal numbers of men and women in occupations as another metric worth studying, as it is associated with higher job satisfaction, better equity in pay and attainment of senior-level positions, and better benefits (Reskin and Roos, 1990; King, 1992; Millward and Woodland, 1995; Preston, 1999).

### Shifting Gender Demographics in Psychology and Associated Changes

Shifts in gender balance and various indicators of occupational health have been examined in psychology because, although it was once a masculinized profession, the field now attracts mostly women (American Psychological Association, Committee on Women in Psychology [APA CWP], 2017). In 1976, for instance, only 33% of new doctorates were women (American Psychological Association [APA CWS], 2009). In the 1980s, women began earning more PhDs in psychology than men (Pion et al., 1996), and by 2017 women were earning 75% of new doctorates in psychology (American Psychological Association, Center for Workforce Studies [APA CWS], 2019). The American Psychological Association (APA) established various task forces to examine the changing gender demographics and their implications within psychology over time.

One of these APA reports (Pion et al., 1996) revealed that although job-level representation was equalizing at that time, gender imbalance within the various subfields of psychology was clear – a trend that continued growing and was documented again with even greater imbalance in the subsequent task force’s report 20 years later (American Psychological Association, Committee on Women in Psychology [APA CWP], 2017). Specifically, health service provider subfields (e.g., clinical, counseling, and school) were and continue to be the fastest growing areas of the field (American Psychological Association, Center for Workforce Studies [APA CWS], 2019), where the proportion of women entering is much higher than men (today about 80% of students in health service provision programs are women), with the gender discrepancy higher than in other areas of psychology (e.g., social, cognitive, and experimental).

The most recent APA task force report about the status of women in psychology concluded that even as women now dominate psychology in terms of numbers, their status, power, and earnings continue to lag behind men (American Psychological Association, Committee on Women in Psychology [APA CWP], 2017). Although many people expected some of the gender gaps would ease as the increasing numbers of women in the field advanced in their careers, several of the indicators of power and prestige were little changed in the 20 years between the APA task force initiative in the mid-1990s and the more recent one in the mid-2010s.

For example, only 18% of APA journal editors are women – just 4% more than 20 years ago (American Psychological Association, Committee on Women in Psychology [APA CWP], 2017). Women are being hired at equal rates to men in academic jobs, but are still underrepresented at the associate and full professor levels and are overrepresented in adjunct, non-tenure-track lecturer, and other temporary positions (American Psychological Association, Committee on Women in Psychology [APA CWP], 2017). And even though women make up 58% of the membership of APA, they represent only 30% of the distinguished fellows of the organization (American Psychological Association, Committee on Women in Psychology [APA CWP], 2017). These patterns have held steady over the past 20 years.

Beyond proportions, power, and status, other measures demonstrated ongoing patterns of change. For instance, there is evidence of decreasing wages in psychology. Data indicate salaries within psychology, compared to doctoral-level earnings in other major science and engineering disciplines, have eroded since the early 1970s (see Pion et al., 1996). The erosion occurred for both genders regardless of their sector of employment – but has impacted women more than men (American Psychological Association, Committee on Women in Psychology [APA CWP], 2017). Further, evidence suggests that the public has serious doubts regarding psychology’s scientific status (Lilienfeld, 2012; Penn, Schoen & Berland Associates, 2008). Psychology is perceived as having made less important contributions to society than biology, chemistry, medicine, and physics (Janda et al., 1998), and notably, the gender pay gap is larger in psychology than in these other fields (American Psychological Association, Committee on Women in Psychology [APA CWP], 2017).

### Income of Psychologists

Relevant data about the hourly rates and income of professionals are available for doctorate holders in general (National Science Foundation [NSF] (2021)), academics consulting in legal settings (Del Rossi and Hersch, 2020), psychologists across settings (American Psychological Association, Center for Workforce Studies [APA CWS], 2017; National Science Foundation [NSF] (2021)), neuropsychologists (Sweet et al., 2021), and psychologists who work in medical school settings (Association of American Medical Colleges [AAMC], 2021), among others. For example, the median annual salary for doctorate holders in general in the U.S. is $104,000, where the gender gap is such that women doctorate holders make $0.78 for every $1.00 that male doctorate holders make (National Science Foundation [NSF], 2021).

In a survey of income involving forensic consultation among academic economists, Del Rossi and Hersch (2020) found that most had experience consulting, and their average hourly rate was $244 with a median of $200. Women charged lower rates than men (an average of $199.20 compared to $260.40, respectively – which corresponds to $0.69 on the dollar for women compared to men). Women were also less likely to have consulted than men, although willingness to consult was similar.

A major report with a nationally representative sample of psychologists describes annual salaries by geographic region, type of degree and position, sector of work, and various demographic characteristics (American Psychological Association, Center for Workforce Studies [APA CWS], 2017). The median annual salary for psychologists in general was $85,000. In professional service positions, psychologists in private practice had the highest median salary at $120,000. Among research psychologists, university-affiliated psychologists had a median salary of $95,000 and private sector research positions at $130,000.

Sweet et al. (2021) surveyed 1,677 neuropsychologists (roughly half of whom practice forensically), finding that the median annual income was $130,900 USD with a gender gap in remuneration where men earn more than women (though the specifics of the gap were not reported). Notably, this study found that neuropsychologists with some forensic involvement had a higher income, at a median of $160,000 whereas the median for non-forensically involved neuropsychologists was $120,000. Hourly fees for adult cases averaged $249.00 for regular clinical neuropsychological evaluations and $347.50 for forensic neuropsychological evaluations (higher for pediatric and lower for lifespan cases).

Many psychologists work in medical school settings, both in practice and in clinical science positions. An analysis of faculty salaries in medical schools showed White men had higher median compensation than anyone else, and that gender was the primary factor driving compensation inequities even though there were also racial and ethnic disparities and intersectional issues (Association of American Medical Colleges [AAMC], 2021). Among medical school faculty with Ph.D. degrees (some of whom are psychologists), women of all races and ethnicities made between $0.68 to $0.89 for every $1.00 White men made. Similarly, the Medical School Psychologist Report (American Psychological Association [APA], 1997) found that men psychologist’s salaries were notably higher than women psychologist’s, regardless of department type, academic rank, and years of experience. In addition, men’s salaries increased steadily with years of experience, but women’s did not.

#### Ongoing Gender Income Disparities in Psychology

In addition to these overall changes to wages and prestige in psychology, there is also evidence of increasing gender disparities within psychology. The American Psychological Association, Committee on Women in Psychology [APA CWP] (2017) task force report shows that women psychologists make an even lower proportion of pay compared to their male colleagues across employment settings than they did in the 1996 task force report (by Pion et al., 1996). For instance, the national average in 1993 was that women psychologists made 85.2% of what men psychologists made, but that proportion dropped to 77.8% in 2010 (American Psychological Association, Committee on Women in Psychology [APA CWP], 2017). These issues are further complicated by findings that female doctoral students in psychology finish graduate school with significantly higher debt than males (American Psychological Association, Committee on Women in Psychology [APA CWP], 2017).

Little data specifically about forensic psychology were available prior to this study. Other studies have found that forensic consulting often generates higher wages than other areas of practice in psychology (e.g., Sweet et al., 2021) as well as in other fields (e.g., Del Rossi and Hersch, 2020), and also that gender disparities are common. Thus, better understanding income patterns in forensic psychology could be useful for career planning and informing equity discussions in the field.

### Gender Equity in Forensic Psychology

Within the realm of forensic psychology, the American Psychology-Law Society (AP-LS)^2^ conducted a survey of members to examine work climate, workload, productivity, professional satisfaction, work/life balance, and leadership issues in the field (American Psychology-Law Society Professional Development of Women Committee [APLS PDW], 2012). Their sample (*N* = 738) was composed of students (52%), non-faculty professionals (26%), and faculty (22%) members of AP-LS, and included both men (35%) and women (65%). Results revealed some discrepancies between men and women’s responses, with the largest discrepancies in academia. They found that female faculty members were less satisfied with their jobs and salaries compared to men, were less likely than men to have held a leadership position within the organization (especially at junior ranks), and were less satisfied with balancing work and family life than men. Similarly, female students reported lower satisfaction than men with regard to support for family and social obligations. These gender differences were not found for psychological professionals who were not in faculty positions. The AP-LS PDW survey provides an important snapshot of various indices of professional satisfaction in the psychology-law field. However, it does not provide information about how the gender composition of the field has changed, nor did it capture income data.

Skeem et al. (2008) reported that almost half of the attendees at workshops provided by the American Academy of Forensic Psychology (AAFP, the educational “arm” of the American Board of Forensic Psychology)^3^ are women, but less than 20% of board-certified forensic psychologists are women. Although women are as likely as men to pass the ABFP exam, fewer women than men apply to take the exam (Skeem et al., 2008). This pattern could be a function of women entering the field more recently than men; however, Skeem et al. (2008) concluded this could not be the only explanation because the pattern remained virtually unchanged in the ten years prior to their report. These findings are important, but here again the broader context of the gender composition of the field remained unclear. The present study addresses this gap in the literature.

The present study examines the shifting gender composition within the field of forensic psychology, including issues of remuneration. It builds on an earlier unpublished study by Neal (2012) that surveyed 351 clinical psychologists with forensic interests in the United States, finding that there was a transition point in the late 1990s before which more men entered forensic psychology and after which more women entered. In exploratory analyses, that study also found trends in the data such that men were more likely to be board certified than women, and more likely to earn PhDs than PsyDs compared to women. The PsyD degree, a practice-oriented degree as opposed to the more scholarly-oriented Ph.D. degree, emerged as a training option in the 1970s and proliferated, eventually overtaking the traditional Ph.D. degree (Norcross et al., 2018). As such, its association with shifting gender proportions in the field is of interest. However, the Neal (2012) study did not ask about income, nor did it include questions about pay negotiations, marital status, children, or family and elder care responsibilities which disproportionately fall to women (National Alliance for Caregiving and the AARP Public Policy Institute, 2015). Each of these variables could be associated with issues of income equity and occupational health.

### The Current Project

We wanted to know whether there are differences in pay and other life and career experiences between men and women practicing in this field, and how the gender composition of the field has changed over time. Little is known overall about how much clinical-forensic psychologists make for their work, even though data about income in other related fields are available. Few studies have examined differences between men and women in terms of when and how they enter the field, obtain training, and practice in clinical-forensic psychology. Gender and professional development trends are important for both individuals and the field as a whole to consider. We thus conducted a brief study to ask people working in the field to share their income information as well as demographic and life-experience questions to analyze relationships between the relevant variables. This information will be useful for career planning and inform discussions of professional equality in the field. To avoid potential anti-trust issues with regard to price-fixing, we take the approach other studies and organizations have taken by presenting data in aggregate form across particular jurisdictions, across sub-areas of the field, across various referral types, and across employment settings (see e.g., American Psychological Association, Center for Workforce Studies [APA CWS], 2017; Sweet et al., 2021).

Informed by previous literature and our own previous findings, we had five *a priori* hypotheses, including (1) men would charge more per hour than women and would make a higher annual total income than women, (2) women would be less likely than men to have ever negotiated over their pay, (3) having a Ph.D. would be disproportionately associated with men and PsyD with women, (4) men would be more likely to be board certified by the American Board of Forensic Psychology (ABFP) than women, and (5) a history of gender imbalance in the composition of the field would emerge. Although more men than women were expected to have entered the field during most of the field’s history, we expected that in the late 1990s the trend would reverse, shifting to more women entering the field (as we saw in Neal, 2012). Finally, we conducted exploratory analyses of the relationships between the variables in the data, such as (1e) how employment type (e.g., institutional setting, private practice) relates to pay, (2e) how gender relates to employment type, (3e) how gender and caretaking responsibilities relate to pay, (4e) how gender relates to postdoctoral experiences, and (5e) how gender, marital status, and children relate to hours worked and pay.

## Materials and Methods

### Procedure

We compiled a master list of potential participants by searching state licensing databases for psychologists licensed in each of the 50 United States using the search capabilities within each state’s directory. Most states provide the names of licensed psychologists in the state with search options for psychologists with particular interests (such as forensic). Some states provide contact information for these psychologists. For those that did not, we enlisted a team of research assistants to search for contact information via professionals’ practice websites. We then sent an email invitation to potential participants with a link to the Qualtrics study with income-related questions followed by demographic questions, which took about 5 min to complete.

Adults who work in a forensic psychology capacity were eligible to participate in this study. “Forensic psychology” was defined as a subfield of psychology in which basic and applied psychological science or scientifically-oriented professional practice is applied to the law to help resolve legal, contractual, or administrative matters (American Psychological Association [APA], 2013; Neal, 2018).

### Participants

We sent an email invitation to 2,041 licensed psychologists with forensic interests; 1,811 of which were delivered.^4^ The response rate was 20.76%, with 376 of the psychologists responding. Of those, some were only partial responses: 311 completed the entire survey.^5^ The respondents were on average 52.80 years of age (*SD* = 13.85), and there were approximately equal rates of women (45.5%) and men (42.0%) in the sample.^6^ The average year in which highest degree was obtained was 1999 (*SD* = 13.18 years), with men’s average year earning highest degree being 1993 (*SD* = 12.85 years) and women’s 2005 (*SD* = 10.63 years; see Table 1 for detailed descriptive statistics about the sample).

**TABLE 1:** Forensic psychologist descriptive statistics with gender comparisons.

	Women (*n* = 171)	Men (*n* = 157)	Total (*N* = 376)
Age (average = mean and standard deviation)	46.70* ± 12.28	59.69* ± 12.21	52.80 ± 13.85
Years of forensic evaluation experience	15.18* ± 9.33	25.87* ± 11.95	20.26 ± 11.91
**Race**			
African-American	3.5	0	1.6
Asian	2.9	0.9	1.6
Hispanic/Latino	4.1	2.5	2.9
White	87.1	91.8	78.2
Other	0.6	2.5	1.3
**Degree**			
Ph.D.	63.9*	75.5*	57.4
Psy.D.	36.0*	24.5*	25.5
J.D./Ph.D.	0.6	5.1	2.4
Other (Ed.D., M.A., J.D.)	1.2	3.7	2.1
Completed a Formal Forensic Postdoc	42.5*	15.6*	29.6
ABFP Certified	27.5	25.9	26.7
Ever Negotiated Pay	61.5*	73.9*	67.5
**Primary Employment Setting**			
Institution or Agency	32.5*	16.1*	21.3
Private	46.2*	65.2*	47.6
University	14.2	7.1	9.3
Other or 1 +	7.1*	11.6*	8.0
**Description of work**			
% forensic-practice-oriented	56.26 ± 33.29	52.83 ± 32.68	54.62 ± 32.99
% clinical (non-forensic) % administrative % teaching % research % consultation	11.55 ± 20.54 14.40 ± 17.97 7.18 ± 7.18 6.71 ± 17.88 3.90 ± 6.81	19.84 ± 20.54 9.17 ± 13.97 6.65 ± 11.85 6.29 ± 14.78 5.21 ± 11.28	15.42 ± 24.57 11.89 ± 16.38 6.91 ± 13.64 6.57 ± 16.47 4.59 ± 9.26
Hours worked per week (mean and SD)	41.09 ± 13.40	40.13 ± 14.93	40.62 ± 14.15
Hourly rate (United States Dollars mean, SD)	258.64* ± 99.73	300.65* ± 112.05	280.23 ± 108.12

The values for race, degree, forensic postdoc, American Board of Forensic Psychology (ABFP) certification, negotiated pay, and primary employment setting are% within gender (e.g., 3.6% women & 0% men in this sample were African-American). For the gender question, no one endorsed “another gender identity,” and 12.5% didn’t answer the question. For the race/ethnicity question, 14.4% of the sample did not respond. For the highest degree question and ABFP certification questions, 12.5% of the sample did not respond. For the forensic postdoc question, 14.6% did not respond. For the description of description of work question, 13.8% did not respond. Hourly rate information is combined across all employment setting types, regions of the country, and across all different types of referral questions *p < 0.05.

They were a forensically-experienced sample, with a little more than 20 years of forensic psychology experience, on average. They spent most of their time on forensic psychological practice activities (54.62% of their monthly time, on average; median = 60%). Eleven percent of participants said they spend all their time on forensic psychological practice activities. About a quarter reported being board certified in forensic psychology by the American Board of Professional Psychology (ABPP). Another 5% were board certified by ABPP in another area (i.e., clinical psychology, clinical neuropsychology, geropsychology). Most were working full-time (82.7%), some part-time (2.9%), and a few retired (1.6%).^7^ Respondents worked in private practice (47.6%), institution or agencies (21.3%; e.g., hospital, prison, court clinic, department of health), university settings (9.3%), and other or more than one setting (8.0%).^8^

Clinicians in 42 of the U.S. states and the District of Columbia participated (states with no respondents included AR, ND, OK, RI, SD, VT, WV, WY). Most were licensed to practice psychology in the U.S. (83.7%), with 2.4% either retired and no longer licensed, a small number of which were licensed outside of the U.S. (e.g., Australia, Canada, New Zealand, the Netherlands), and a small number in the process of becoming licensed.^9^

About a quarter of respondents completed a formal postdoctoral fellowship in forensic psychology, with many from programs currently accepted by the American Board of Forensic Psychology (American Board of Forensic Psychology, Inc. [ABFP], 2020) for experience waivers to their 5-year rule (i.e., in order by descending frequency of respondents: University of Massachusetts Medical School; Central State Hospital in Virginia; Patton State Hospital in California; Minnesota State Operated Forensic Services; Augusta University/East Central Regional Hospital in Georgia; Medical University of South Carolina; Mendota Mental Health Institute and Sand Ridge Secure Treatment Center in Wisconsin; Center of Excellence for Children, Families, and the Law in Massachusetts; Bridgewater State Hospital in Massachusetts; Emory University School of Medicine in Georgia; Northwest Forensic Institute in Oregon; Walter Reed National Military Medical Center; and Western State Hospital in Washington). More than two dozen other sites of forensic postdoctoral training were reported, including university settings (e.g., University of California, Los Angeles; University of Rochester Medical School in New York; University of Arkansas Medical School; University of Southern California; University of Virginia; University of Washington), hospital and secure hospital settings (e.g., Saint Elizabeth’s Hospital in Washington DC; Wyoming State Hospital; U.S. Medical Center for Federal Prisoners in Missouri; Florida State Hospital; Springfield Hospital in Maryland; Metropolitan State Hospital in California), and various other settings, including five respondents who did postdocs in private practice settings.

Because we thought that variables like marital status, children, and care-taking responsibilities might affect income, we also asked about these things. The majority of the sample reported being married (64.1%; 8.0% never married, 6.6% divorced, 2.9% widowed, 0.8% separated),^10^ and a little more than half had children (57.2%; 1.3% reported “it’s complicated” with step-children and other situations).^11^ A significant portion reported having had, at any point in their career, significant care-taking responsibility for others (e.g., children, elders, sick family members) for an extended period of time (41.5%; 1.9% reported “it’s complicated” with Covid-19 impacts and other issues detailed).^12^

## Results

**FIGURE 1:**
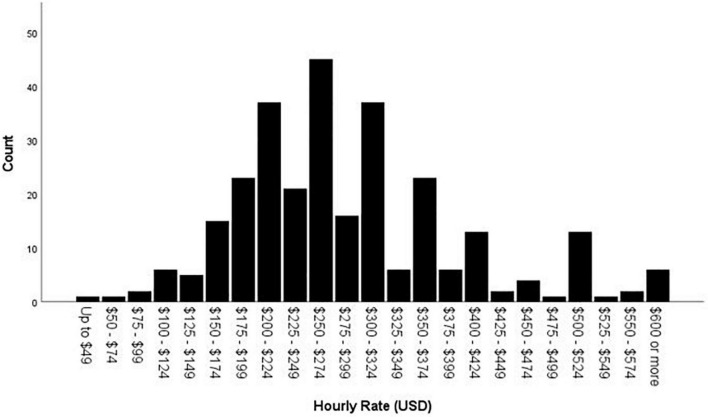
Aggregate hourly rate (USD).

**FIGURE 2:**
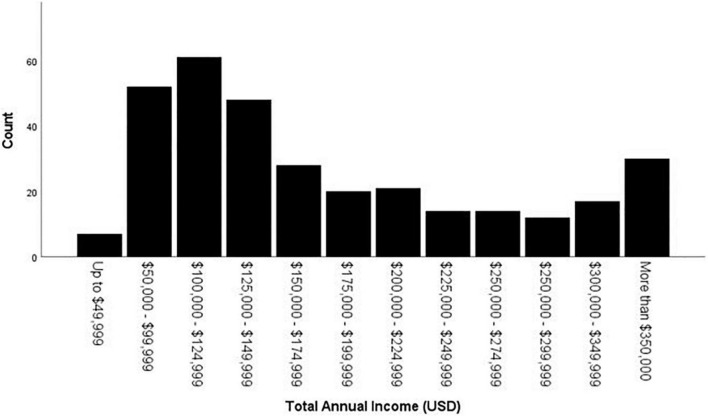
Total annual income (USD).

As can be seen in bottom row of Table 1 and Figure 1, the average hourly rate charged by psychologists for forensic work across all types of referral questions, employment settings, and regions of the country is $280.23 (US Dollars; median and mode = $250). Table 2 shows the hourly rate per gender across regions of the United States. Total annual income (own income, not including partner or family income) in US Dollars had a median of $125,000 - $149,999 and a mode of $100,000 - $124,999 (see Figure 2).

**TABLE 2:** Hourly rate (USD) of forensic psychologists across U.S. regions.

	Women (*n* = 147)	Men (*n* = 139)	Total (*N* = 286)
Midwest (mean ± standard deviation)	216.05 ± 88.71	262.05 ± 59.79	237.90 ± 78.91
Northeast	245.41 ± 93.91	306.29 ± 105.18	278.19 ± 103.79
South	280.42 ± 102.60	304.53 ± 104.26	292.20 ± 103.53
West	266.07 ± 105.08	312.54 ± 131.29	290.36 ± 121.09
Other	247.14 ± 69.97	288.09 ± 141.34	272.17 ± 117.91

*Ninety participants did not respond to the question about hourly rate. Regions are defined as Midwest = (the states of OH, MI, IL, IN, WI, MN, IA, MO, ND, SD, NE, KS), Northeast = (MA, NH, VT, MA, CT, RI, NY, NJ, PA, ME), South = (TX, OK, AR, LA, MS, AL, GA, FL, TN, KY, WV, VA, NC, SC, MD, DE), West = (AK, HI, WA, OR, CA, NV, ID, UT, AZ, NM, CO, WY, MT), and Other included regions outside of the 50 states (5 participants were in this group). Our reporting by region while collapsing across state jurisdictions is consistent with other sources, such as Sweet et al.’s (2021) reporting of neuropsychologists’ income and the American Psychological Association, Center for Workforce Studies [APA CWS] (2017) reporting on psychologists’ income, which is based on National Science Foundation data for income across professions (which is also available by region).*

**FIGURE 3:**
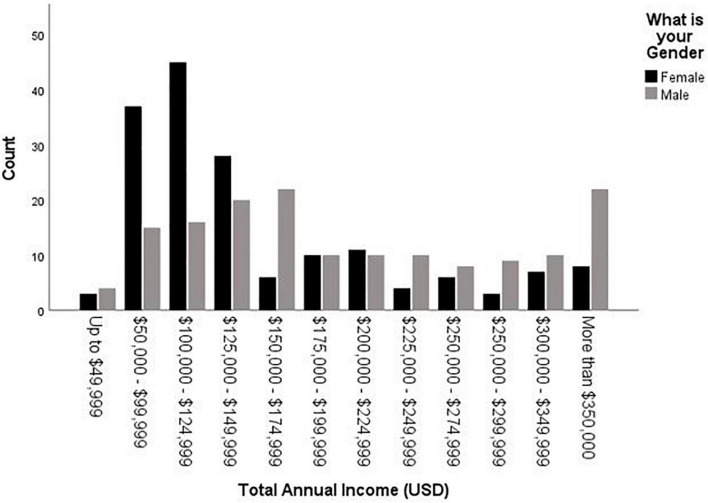
Total annual income (USD) by gender.

Our first hypothesis was largely supported. Men charged significantly more per hour than women, *F*(1,284) = 11.17, *p* < 0.001, *η_*p*_^2^* = 0.04 (for descriptives, see Table 1); however, once controlling for years of experience, those gender differences were no longer significantly different, *F*(1,263) = 1.16, *p* = 0.28, *η_*p*_^2^* = 0.004. In addition, as expected, men’s total annual income was significantly higher than women’s, Wald χ*^2^* = 29.16, *p* < 0.001. Figure 3 shows total annual income by gender, where the median for men is $175,000 - $199,000 (vs. $100,000 - $124,999 for women) and mode for men is $150,000 - $174,999 (vs. $100,000 - $124,999 for women). Once adding in controls for years of experience and number of hours worked per week, total annual income was still higher for men, Wald χ*^2^* = 7.96, *p* = 0.005. Overall (with no statistical controls), women forensic psychologists earn $0.83 for every $1.00 men make.

Partially consistent with hypothesis 2, women were less likely than men to have ever negotiated the amount of their pay, χ*^2^* = 5.65, *p* = 0.017 (see Table 1). However, once controlling for years of experience, there was no longer a significant difference (OR = 1.24, 95% CI = 0.72 – 2.14, *p* = 0.50). Similarly, regarding hypothesis 3, having a PhD was disproportionately associated with men compared to women, and having a PsyD was disproportionately associated with women compared to men, χ*^2^* = 5.65, *p* = 0.017. However, once controlling for years of experience, given the increasing prevalence of the PsyD degree in the field over time, there was no longer a significant difference, (OR = 0.88, 95% CI = 0.50 – 1.56, *p* = 0.67). Hypothesis 4 was unsupported: in this sample, men were no more likely to be board certified than women, χ*^2^* = 0.99, *p* = 0.75 (see Table 1).

Consistent with hypothesis 5, we found the gender composition of the field has reversed itself. To examine change in the gender composition of the field over time, we used Gross’s (1968) Index of Dissimilarity. This index allows for quantifying the degree of gender imbalance within an occupation and tracing the degree of difference over time. When looking at multiple different occupations, the statistic calculated through this index can be interpreted as “the percentage of women (or men) that would have to change jobs in order for the distribution of both to be the same” (Preston, 1999, p. 612). In our case, we look at a single occupation over a range of time increments. In this context, the statistic can be interpreted as the percentage of women (or men) that would have had to enter the field at a different time than they had in order for the distribution of both to be the same at that time point.

**FIGURE 4:**
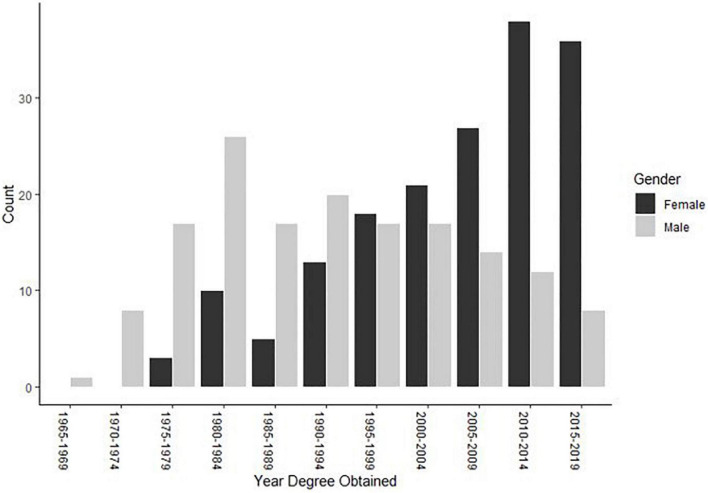
Year terminal degree received by raw count within gender.

To do this analysis, the continuous variable “year since terminal degree was received” was categorized into five-year increments (i.e., percentage of men and women receiving their terminal degree in 1965-1969, 1970-1974, 1975-1979, 1980-1984, 1985-1989, 1990-1994, 1995-1999, 2000-2004, 2005-2009, 2010-2014, 2015-2019). The gender proportions within each decade are depicted in Table 3 and the numbers of men and women earning their degrees within each decade are depicted in Figure 4. The Index of Dissimilarity was calculated to explore the overall proportional gender distribution across those increments of time (see Table 3). The overall index was 38.9%, meaning that about a 39% change in gender distribution would need to have occurred for equal representation. The value of the overall index, however, masked the change that occurred over time. Therefore, separate indexes were calculated before and after 2000 (see Table 3 and Figure 4). The discrepancy was weighted more heavily toward men entering the field before 2000, whereas the discrepancy since 2000 was weighted toward more women entering the field. Prior to 2000, 19.4% more women were needed for equal distribution, but since 2000, 19.5% more men were needed.

**TABLE 3:** Index of gender dissimilarity within forensic psychology over time.

Year terminal degree received	Women (% of women)		Men (% of men)	Abs. Diff.	Index (Sum of Abs. Diff./2)
Index over 5-year increments					
1965-1969 1970-1974 1975-1979	0.0 0.0 1.8	< <	0.6 5.1 10.8	0.6 5.1 9.0	
1980-1984 1985-1989	5.8 2.9	< <	16.6 10.8	10.8 7.9	
1990-1994 1995-1999	7.6 10.5	< ≈	12.7 10.8	5.1 0.3	
2000-2004 2005-2009 2010-2014 2015-2019	12.3 15.8 22.2 21.1	> > > >	10.8 8.9 7.6 5.1	1.5 6.9 14.6 16.0	
Sum =				77.8	
					***38.9***
Index calculated before vs. after 2000					
Sum Prior to 2000 =	28.6%	<	67.4%	38.8	
					***19.4***
Sum Since 2000 =	71.4%	>	32.4%	39.0	
					***19.5***

*Abs Diff = Absolute Difference. Index = Sum of Absolute Differences divided by two (Duncan and Duncan, 1955). The values for men and women within each decade are represented by proportions within gender as opposed to raw counts to control for differences in group sizes and changes in the size of the occupation or entry year over time. The value of the index is interpreted as the percent of gender that would have to change entry year in order to have equal percentage distributions across the five-year increments (Gross, 1968; Preston, 1999).*

### Exploratory Analyses

**FIGURE 5:**
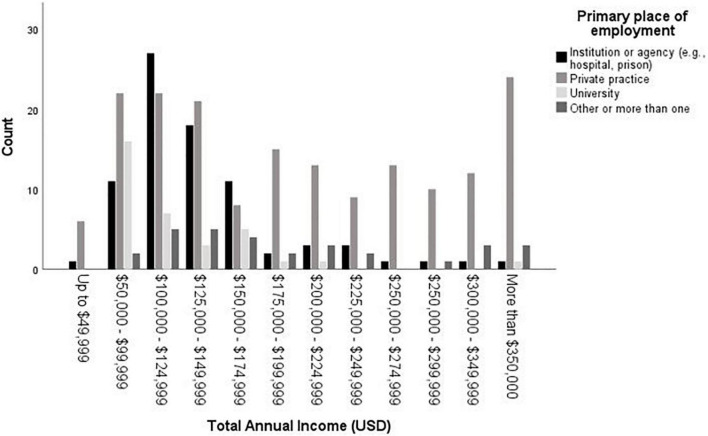
Total annual income (USD) by primary place of employment.

We further analyzed the data with exploratory analyses. Employment type related to pay, χ*^2^*(3) = 42.64, *p* < 0.001 (see Figure 5). Specifically, people who worked primarily in private practice (median = $175,000 - $199,999, mode = more than $350,000) made significantly more than those who worked primarily in institutions (e.g., prisons, hospitals; median = $125,000 - $149,000, mode = $100,000 - $124,999), Wald χ*^2^* = 19.69, *p* < 0.001. People who worked in private practice also made significantly more than those who worked primarily in university settings (median = $100,000 - $124,999, mode = $50,000 - $99,999), Wald χ*^2^* = 28.89, *p* < 0.001. Private practice did not differ significantly from people who worked in “other or more than one setting” (median = $150,000 - $174,999, mode = $100,000 - $124,999), Wald χ*^2^* = 0.02, *p* = 0.89.

Consistent with the findings of LaDuke et al. (2020), employment setting varied by gender: the odds of men being in private practice was nearly three times that of women as compared to institutional employment (OR = 2.85, 95% CI = 1.63 – 4.98), and men had more than three times the odds of being employed multiple settings (e.g., private practice in addition to institutional or university employment, other situations) rather than in an institutional setting (OR = 3.30, 95% CI = 1.38 – 7.88), controlling for all other settings. No differences in university employment emerged. However, once adding year of highest degree into the model, gender was no longer significant. Most of the model variance was attributable to recency of highest degree, with each 1 year increase in year degree obtained (i.e., each year their degree is more recent) being associated with about a 9% reduced likelihood of being in private practice as compared to an institutional setting (OR = 0.94, 95% CI = 0.78 - 2.73), and nearly 10% reduced likelihood of employment in multiple settings as compared to institutional setting (OR = 0.96, 95% CI = 0.921 - 0.995).

We expected caretaking responsibilities might relate to gender and pay in systematic ways; however, there were no gender differences in significant caretaking responsibilities for others (e.g., children, elders, sick family members), χ*^2^* = 0.19, *p* = 0.73. We also wanted to see how gender and caretaking responsibilities would interact to affect pay: although the main effect of gender on income emerged as before (Wald χ*^2^* = 21.30, *p* < 0.001), no main effect of caretaking on income emerged (Wald χ*^2^* = 0.37, *p* = 0.54), with no interaction between gender and caretaking on income (Wald χ*^2^* = 1.85, *p* = 0.17).

Gender systematically related to postdoctoral experiences, such that women (42.51%) were much more likely than men (15.58%) to have completed a formal postdoctoral fellowship in forensic psychology, χ*^2^* = 27.89, *p* < 0.001 (see Table 1). One explanation could be that women entered the field more recently, on average, than men and postdocs may have become a more common training experience in more recent years. However, this relationship held even when controlling for year of highest degree, Wald χ*^2^* = 7.71, *p* = 0.005, OR = 2.28.

Finally, we sought to explore the how gender, marital status, and children relate to number of hours worked. No main effects or interactions emerged, model *F*(16, 280) = 0.50, *p* = 0.95, *η_*p*_^2^* = 0.028. Gender and children did relate to income; specifically, main effects emerged for gender (Wald χ*^2^* = 4.10, *p* = 0.043) and children (Wald χ*^2^* = 6.89, *p* = 0.009), but no interaction emerged (Wald χ*^2^* = 0.11, *p* = 0.74). Men made more money than women even when controlling for whether or not people had children, and people with children made more money than people without children while controlling for gender.

## Discussion

The purpose of this study was to compile descriptive information about forensic psychologists to compare the income, demographics, and life experiences of men and women and to examine some indicators of professional equality in the field. We sought to answer whether there are gender differences in pay and other life and career experiences between men and women, and how the gender composition of the field has changed over time. Providing information about pay equity and understanding reasons for inequities has the potential to reduce gender gaps and improve professional parity.

As far as we are aware, these are the first aggregate data available about the income of forensic psychologists. The findings presented here are generally consistent with other sources of data regarding gender and income disparities between men and women in psychology (e.g., American Psychological Association, Committee on Women in Psychology [APA CWP], 2017; National Science Foundation [NSF] (2021), Sweet et al., 2021). These data are useful about reflecting on gender equality, as well as to inform students and practitioners about the potential future earnings of different choices they might make during their training and job searches, as people “need to be able to find [information like this]” in order “for it to have influence” (Holpuch, 2022, p1).

Findings revealed men have been practicing longer on average than women, but that gender trends in the field are changing. Although the field used to be heavily dominated by men, in recent years forensic psychology has been attracting more women than men. As of about the year 2000, men were no longer entering the field in greater numbers than women. The trend of increasing proportions of women in the field is even higher in the current project than in Neal’s (2012) analysis, as the first cohort of forensic psychologists in the field who earned their highest degrees in the 1950s and 1960 (who were mostly men) have retired since that 2012 project.

Forensic psychology is not alone in its process of feminization. Psychology in general (Pion et al., 1996; American Psychological Association, Committee on Women in Psychology [APA CWP], 2017), along with most other professions and scientific disciplines, as well as the pipeline in higher education itself (Leathwood and Read, 2009; Field, 2021), has undergone dramatic shifts in gender composition. It appears that the pattern of changing gender demographics in forensic psychology is similar to the pattern occurring in psychology as a whole, albeit at a slower pace. For instance, the APA task force on gender demographics in psychology as a whole identified the 1980s as the tipping point decade (Pion et al., 1996), whereas the data here identify the late 1990s as the tipping point when more women than men began entering forensic psychology (refer to Table 3 and Figure 4). The slower pace of shifting gender composition within our subfield may be due in part to the higher earning power within forensic psychology compared to other psychology subfields (e.g., Sweet et al., 2021), as well as fewer managed care restrictions in forensics as compared to other health-service psychology subfields (Otto and Heilbrun, 2002).

Although more women are entering the field compared to earlier decades, differences exist in some indicators of professional equality. Men earn more than women annually in this field, even while controlling for years of experience and number of hours worked per week, consistent with broader trends in American society and with other doctorate-level professions (see e.g., Lo Sasso et al., 2020; Association of American Medical Colleges [AAMC], 2021). Some of that difference may be explained by employment setting, with people in private practice settings (who are more likely to be men) making more money than people employed in institutional settings (who are more likely to be women). Additionally, women are more likely to complete a formal forensic training postdoc than men, even controlling for year of highest degree. The implication of this finding is that women may go through more years of training and expend more resources to enter this field than men. Previous studies reported women are less likely to hold the highest positions of leadership in the field, are less likely to be awarded the field’s most prestigious honors, are underrepresented in senior career positions, and report lower levels of professional and work/life balance satisfaction compared to men (Skeem et al., 2008; American Psychology-Law Society Professional Development of Women Committee [APLS PDW], 2012). These findings merit attention and the generation of creative solutions.

Despite those more concerning findings, other indicators of professional equality were more promising in these data. For instance, women were just as likely to be board certified as men, and women and men work similar number of hours per week. No differences in self-reported significant care-taking responsibility for others (e.g., sick family members, elders, children) emerged, even though more men than women were married and had children. Men were more likely than women to have negotiated their pay, with career-long implications for income (Corbett and Hill, 2012), but that difference in negotiation went away once controlling for years of experience. Thus, perhaps people are more likely to negotiate pay later in their careers than they are earlier. Although type of highest degree was associated with gender, such that having a PhD was disproportionately associated with men and PsyD with women (with debt load implications, as PsyDs accumulate more student debt on average than PhDs; Cantone et al., 2021), this pattern went away once years of experience was added to the analysis. This finding suggests that PsyDs are relatively more common now for both men and women as compared to previous decades in which the PhD degree was more standard.

To supplement this discussion, we explored how women’s leadership and award representation might have changed over time in the field. Specifically, we updated some of the analyses by Skeem et al. (2008) by examining publicly available data current through 2021. The field’s Distinguished Contributions to Psychology and Law Award has been awarded to more men (28) than women (7), but the ratio has improved over time (i.e., 93.3% men prior to 2000, 70% men since 2000). The field’s Saleem Shah Early Career Award has been awarded to 15 men and 14 women, with more men in the earlier years of the award (1995 – 2005), equal numbers of men and women from 2006 through 2015, and more women in recent years (since 2016). More men (11) than women (8) have been president of the American Board of Forensic Psychology, though the representation here has varied across time as well, with many more men in earlier years and relatively more women in more recent years. These patterns suggest that women’s representation is improving in leadership positions and in prestigious honors as compared to previous decades.

### Limitations and Future Directions

If we had the opportunity to do this survey again, we would ask some questions differently. For example, we asked an open-ended question about flat fees for forensic evaluations, but it yielded messy and difficult-to-interpret data that we do not report. We also did not distinguish between public or private counsel referrals, which in free response comments people noted affects their rate. In addition, we would increase the upper limit for income range on the survey. We used an income-survey approach with ordinal scale bins ranging up to $350,000 +. Eight percent of the sample selected that highest bin, and some of the comments included things like, “I think you should increase the upper limit for income range on this survey. Mine is well above the top choice [of $350,000] and I imagine others’ income may be above as well” and “The survey would be more informative if the income brackets went higher, i.e., to $700K.” We also would be clearer about asking about taxable income as opposed to fees collected, as these may differ especially for private practitioners.

We focused primarily on psychologists in the United States, and as such, the findings of this project may not generalize well to other countries. We hope future work will build on this study with samples from other countries too to generate a cohesive body of work from which the field can understand broader global trends. In addition, the historical context of this project is worth noting. A few people mentioned the impacts of Covid-19 on their income, which should be considered as the timing of this survey was during the Covid-19 pandemic (data were collected in July 2021). For example, one respondent commented, “My income was down ∼25% due to Covid” and another said, “My income for 2021 is likely to be lower due to stress about my dad and Covid-19 in general.”

We did not ask questions about satisfaction about work and work-life balance that could have been examined in relation to this income and other work-related information. However, LaDuke et al. (2020) recently investigated these issues through the American Psychology-Law Society. When those data are available, they will further the conversation about these issues in the field. There are several other important indices of equality that future researchers might explore, including the relative achievements of men and women in the field, leadership positions held, various performance indicators (such as promotions, publications, honors received), challenges faced by men and women, and their workload, productivity, and general well-being and satisfaction both with their careers and with work-life balance issues.

If a stable gender balance is a worthwhile goal, can it be achieved in the field? What can we do to encourage equal numbers of men and women to seek entry into a forensic psychology training program at the application stage to improve our chances of having approximately equal representation in the graduate education pipeline? Undergraduate and graduate institutions might investigate reasons why people decide to pursue these degrees (e.g., Marulanda and Radtke, 2019), and encourage both men and women to apply to psychology-law related graduate programs, as equal representation in graduate acceptance rates and the educational pipeline might best be accomplished from equal representation in graduate program applications. Attention to parity across important aspects of work in this field will continue to be important, even while considering how to better encourage a stable gender balance in the field.

In their Action Plan, the American Psychological Association, Committee on Women in Psychology [APA CWP] (2017) encouraged psychologists to identify and initiate strategies to maintain and enhance the professional, scientific, and economic status of psychology as well as to continue to be responsive to society’s needs. They encouraged efforts to inform policymakers about how psychology’s expertise and contributions can effectively be used and to collaborate with other disciplines to address society’s diverse and interdependent issues (see also Kupferschmidt, 2022). Based on their analysis of the future of women in psychology, Gruber et al. (2021) suggested that women at all professional levels learn negotiation skills, including finding information about the income of comparable peers. These recommendations continue to have merit and apply to our subfield of forensics.

In addition, the 2017 task force recommended APA advocate for gender equity, such as through state and federal policy to encourage salary transparency and monitoring progress. In an effort to reduce the gender pay gap, other countries including the United Kingdom, Australia, and Germany now require companies and organizations to report on their pay practices for men and women each year (Holpuch, 2022), which the U.S. could require but does not (yet). Similarly, others have highlighted that income equity could benefit from better understanding its systemic underpinning and intersectional phenomena, and working in good faith to address those issues such as through public reporting of income data, equity initiatives to close pay gap, and tracking and sharing progress for accountability (e.g., Association of American Medical Colleges [AAMC], 2021; Gruber et al., 2021). The data in this paper are consistent with the spirit of this recommendation. We hope people find these data useful and interesting, and that students thinking about entering the field and people already working in the field can use these findings to make informed decisions about what to do with their careers.

## Data Availability Statement

The datasets presented in this study can be found in online repositories. The names of the repository/repositories and accession number(s) can be found below: More information about this project is available on the Open Science Framework (RRID:SCR_003238): https://osf.io/5pzyt/, including portions of the data that we could share (we could not share it all due to concerns about privacy, which we discuss in the codebook explaining the data we posted), materials, other relevant files, the summary of results shared with participants in summer 2021, and a preprint version of the manuscript.

## Ethics Statement

The studies involving human participants were reviewed and approved by the Institutional Review Board at Arizona State University. Written informed consent for participation was not required for this study in accordance with the national legislation and the institutional requirements.

## Author Contributions

TN and EL: conceptualization, methodology, formal analysis, data curation, writing, and visualization. TN: investigation. Both authors contributed to the article and approved the submitted version.

## Conflict of Interest

The authors declare that the research was conducted in the absence of any commercial or financial relationships that could be construed as a potential conflict of interest.

## Publisher’s Note

All claims expressed in this article are solely those of the authors and do not necessarily represent those of their affiliated organizations, or those of the publisher, the editors and the reviewers. Any product that may be evaluated in this article, or claim that may be made by its manufacturer, is not guaranteed or endorsed by the publisher.

## Footnotes

1 In this paper, all currency with the $ notation is in United States Dollars (USD).

2 AP-LS, division 41 of the American Psychological Association, is an interdisciplinary organization composed of people devoted to practice, public service, and scholarship in psychology and law, many of whom identify as clinical-forensic psychologists (see http://www.ap-ls.org/).

3 The American Board of Forensic Psychology (ABFP), an affiliated member of the American Board of Professional Psychology (ABPP), is a consumer-protection organization that awards specialty clinician certification to psychologists who satisfactorily complete the diplomate requirements (see http://www.abfp.com/).

4 Of the 2,041 emails originally sent, 198 bounced, there were 25 duplicates, and 7 failed = successfully delivered to 1,811 psychologists.

5 We excluded participants only for those analyses for which they had missing data (i.e., the participants were still included in the dataset even with partial data, and were included in results when the data were available for the given variables of interest for each analysis).

6 No one endorsed “another gender identity,” and 12.5% didn’t answer the gender question.

7 12.5% did not to respond to this question. Of those that responded, 96.5% of women were working full time (vs. 92.4% men), 1.8% were part-time, and 1.2% were retired (vs. 5.1% and 2.5% men, respectively).

8 13.8% of the sample did not respond to this question.

9 12.5% of the sample did not to respond to this question. Of those that did respond, 94.3% of men and 92.3% of women reported being licensed in the U.S.

10 17.6% of the sample did not respond to this question. More men (87.7%) than women (68.9%) reported being married, and more women than men reported being never married (14.0% vs. 4.8%), divorced (10.4% vs. 5.5%), widowed (4.9% vs. 2.1%), and separated (1.8% vs. 0%).

11 17.3% of the sample did not respond to this question. More men (81.6%) than women (57.9%) reported having children.

12 18.4% of the sample did not respond to this question. Both men (50.7%) and women (53.2%) reported having had significant care-taking responsibilities.
